# The Functional Neuroanatomy of Reading Intervention

**DOI:** 10.3389/fnins.2022.921931

**Published:** 2022-06-16

**Authors:** Jeremias Braid, Fabio Richlan

**Affiliations:** Department of Psychology, Centre for Cognitive Neuroscience, Paris Lodron University of Salzburg, Salzburg, Austria

**Keywords:** reading intervention, reading disability, developmental dyslexia, neuroimaging, review

## Abstract

The present article reviews the literature on the brain mechanisms underlying reading improvements following behavioral intervention for reading disability. This includes evidence of neuroplasticity concerning functional brain activation, brain structure, and brain connectivity related to reading intervention. Consequently, the functional neuroanatomy of reading intervention is compared to the existing literature on neurocognitive models and brain abnormalities associated with reading disability. A particular focus is on the left hemisphere reading network including left occipito-temporal, temporo-parietal, and inferior frontal language regions. In addition, potential normalization/compensation mechanisms involving right hemisphere cortical regions, as well as bilateral sub-cortical and cerebellar regions are taken into account. The comparison of the brain systems associated with reading intervention and the brain systems associated with reading disability enhances our understanding of the neurobiological basis of typical and atypical reading development. All in all, however, there is a lack of sufficient evidence regarding rehabilitative brain mechanisms in reading disability, which we discuss in this review.

## Introduction

Reading acquisition or learning to read is a complex endeavor requiring the integration of orthographic, phonological, and semantic information about written words together with knowledge of spoken language and conceptual knowledge. In a considerable number of cases, however, children struggle with the acquisition of foundational reading skills—a condition known as reading disability (RD) or developmental dyslexia. Specifically, RD is characterized by severe and persistent problems in reading acquisition.

In children with RD, performance in standardized reading tests is significantly below the age-expected norm. In addition, people affected by RD often present a mixture of different manifestations of problems in diverse aspects of literacy including reading fluency, accuracy, comprehension, and/or spelling (e.g., Lyon et al., 2003). Importantly, the difficulties cannot be explained by problems regarding intelligence, motivation, vision, or educational environment. Finally, these difficulties markedly impair academic achievement or activities in everyday life requiring reading skills (American Psychiatric Association, 2013; World Health Organization, 2016).

The present mini-review aims to concisely summarize the literature on neuroplasticity following reading intervention and to relate it to the functional neuroanatomical models of reading and RD. For that purpose, we review the systematic findings regarding brain mechanisms underlying reading improvements following behavioral intervention for RD (covering multiple rehabilitation techniques). This includes evidence of neuroplasticity concerning functional brain activation, brain structure, and brain connectivity. Finally, we discuss limitations, open issues, and future perspectives in order to pave the way for further progress in this field.

## The Functional Neuroanatomy of Reading and Reading Disability

### Functional Brain Activation

During the last years, there has been considerable progress in understanding the neurocognitive and neurobiological mechanisms underlying reading and RD. Using brain imaging techniques such as functional magnetic resonance imaging (fMRI), electroencephalography (EEG), and magnetoencephalography (MEG), studies have largely converged on the brain circuits involved in typical and atypical reading. Specifically, the functional neuroanatomical model of typical reading involves a predominantly left-lateralized network including occipito-temporal (OT), temporo-parietal (TP), and frontal language regions (e.g., Dolan et al., 1997; Paulesu et al., 2000; Cattinelli et al., 2013; Martin et al., 2015; Schuster et al., 2016; Chyl et al., 2021).

With respect to RD, qualitative reviews and quantitative meta-analyses have identified altered brain activation in atypical readers during reading or reading-related tasks in this left-hemisphere network. In particular, the most consistent finding across studies was underactivation in people affected by RD compared with their age-matched peers in the left ventral OT cortex (fusiform gyrus, FFG and posterior inferior temporal gyrus, ITG), the left posterior middle and superior temporal gyrus (MTG and STG), and the left inferior frontal gyrus (IFG) (e.g., Paulesu et al., 2001, 2014; Maisog et al., 2008; Richlan et al., 2009, 2011; Martin et al., 2016).

Underactivation of the left hemisphere reading network—in particular the language-universal dysfunction of the left ventral OT cortex—most probably reflects the phonological speed deficit characteristic of RD. This is in line with evidence showing that in typical readers the ventral OT cortex subserves both lexical whole-word recognition and sublexical serial decoding (e.g., Richlan et al., 2010; Schurz et al., 2010; Wimmer et al., 2010). Conversely, overactivation in atypical compared with typical readers was identified in the left precentral cortex and the bilateral frontal striatum (including caudate and putamen), perhaps reflecting overreliance on sub-vocal articulatory-based reading processes (Richlan, 2012, 2014, 2020; Hancock et al., 2017).

There is an increasing number of hints on the existence of additional functional activation abnormalities in cortical, sub-cortical, and cerebellar regions in RD (e.g., Danelli et al., 2012; Mascheretti et al., 2017; Alvarez and Fiez, 2018; De Vos et al., 2020), but this has not yet been evidenced by objective quantification through systematic meta-analysis. The reasons for this absence most probably lie more in methodological limitations of the meta-analyses themselves, than in the primary studies. Obviously, any alterations in functional brain activation strongly depend on the in-scanner tasks and baseline conditions, as well as several other experimental considerations related to stimulus types, presentation modalities, instructions, sample sizes, analytical techniques, statistical thresholds and last but not least diagnosis/inclusion criteria for the RD groups (see section “Limitations, Open Issues, and Future Perspectives”).

### Gray and White Matter Structure and Connectivity

Quantitative meta-analyses on gray matter (GM) structural abnormalities in RD as investigated by means of voxel-based morphometry showed a similar picture, with limited convergence across studies (for an in-depth discussion see Ramus et al., 2018). The most robust and consistent finding was GM volume reduction in atypical compared with typical readers in the right STG and the left superior temporal sulcus (STS), but only about half of the primary studies contributed to these meta-analytic clusters (Linkersdörfer et al., 2012; Richlan et al., 2013; Eckert et al., 2016).

Across different languages, the left STS is assumed to play an important role in the integration of auditory and visual information (e.g., Van Atteveldt et al., 2004; Blomert, 2011; Holloway et al., 2013; Richlan, 2019). Therefore, in typical reading acquisition, it plays a pivotal role during self-reliant learning processes based on serial grapheme-phoneme conversion. The STG/STS GM volume reduction found in RD might be related to a deficit in this sublexical self-teaching reading strategy, specifically in the development of a brain system for efficient interactive processing of auditory and visual linguistic inputs (Blau et al., 2010).

With respect to white matter (WM) structure and connectivity, the major pathways supporting skilled reading are found in left TP areas and in posterior callosal tracts including the superior longitudinal fasciculus (including the arcuate fasciculus, AF), occipital and temporal callosal fibers, and corona radiata fibers passing through the posterior limb of the internal capsule (Ben-Shachar et al., 2007). In RD, these pathways have been identified with lower fractional anisotropy values (indicating reduced structural integrity) in diffusion tensor imaging (DTI) studies. A prime candidate fiber tract most consistently associated with RD is the left AF, which connects left TP and left frontal language regions (Silani et al., 2005; Vandermosten et al., 2012; Dehaene et al., 2015). Additional findings point to deficits in visual thalamo-cortical connections (Müller-Axt et al., 2017).

The left AF was reported to be among the first brain circuits to anatomically change during reading acquisition. Specifically, learning to read has been shown to be accompanied by an increase in fractional anisotropy (FA) and a decrease in perpendicular diffusivity (PD) (reflecting a microstructural improvement) of this fiber tract (Thiebaut de Schotten et al., 2012; Yeatman et al., 2012). Based on these findings, the left AF is assumed to play a crucial role, especially during the early stages of literacy development by supporting letter-speech sound integration and grapheme-phoneme coding, which, in turn, is required for self-reliant phonological word decoding in beginning readers (Richlan, 2019).

### Functional Connectivity and Integration

Besides structural connectivity by means of DTI, studies on functional and effective connectivity provide interesting insights into how brain regions interact with each other in order to support skilled reading (e.g., Mechelli et al., 2005; Schlaggar and McCandliss, 2007; Vogel et al., 2013; Carreiras et al., 2014; Schurz et al., 2014). Put simply, in typical readers, left OT, TP, and IFG regions are functionally connected, whereas in RD this functional coupling is disrupted, either as a cause or consequence (or both) of reading difficulties. Reduced functional connectivity in RD within the typical left-hemisphere reading network was found both during reading and reading-related tasks (e.g., Paulesu et al., 1996; Van der Mark et al., 2011; Boets et al., 2013; Olulade et al., 2015; Cao et al., 2017; Morken et al., 2017) as well as in the absence of a task, that is, during rest (e.g., Koyama et al., 2013; Schurz et al., 2015).

The idea that RD results from disrupted connections between brain regions supporting vision and brain regions supporting language has been around for decades (Geschwind, 1965a,b; Paulesu et al., 1996). As evidenced by modern-day neuroimaging, this disruption of brain systems might reflect the characteristic visual-verbal speed deficit in the behavioral manifestation of RD, which, in turn, is attributed to inefficient access from letters to speech sounds. This deficit in RD was hypothesized to underlie the universal reading speed impairment across languages (Wimmer, 1993; Ramus and Szenkovits, 2008; Blomert, 2011; Richlan, 2019). As pointed out in the following section, behavioral interventions for people affected by RD often focus on letter-speech sound integration and on linking sub-lexical and lexical orthographic and phonological information (e.g., Fraga González et al., 2015).

## Reading Intervention and Neuroplasticity

### Behavioral Effects of Reading Intervention

RD poses a significant burden for those affected (American Psychiatric Association, 2013; World Health Organization, 2016). Fortunately, many studies have shown that reading intervention can be beneficial for people with RD (e.g., Wanzek et al., 2018). By and large, explicit phonics instruction can be regarded as the gold standard in reading intervention programs due to its beneficial effects on a large amount of RD people (Galuschka et al., 2014). This includes interventions aimed at teaching (a) letter-speech sound correspondences, (b) decoding strategies that involve blending or segmenting individual letters or phonemes, and (c) dividing spoken or written words into syllables or onsets and rimes.

Systematic meta-analyses revealed moderate effect sizes regarding improvement in reading ability after reading intervention (Wanzek et al., 2013, 2016, 2018). The examined intervention programs, however, differed significantly in a number of aspects such as skills targeted, duration, intensity, modality, and group size. In addition, marked individual differences between participants within particular studies impede generalization. Therefore, specific conclusions on the efficacy of intervention programs must be drawn with caution.

### Brain Effects of Reading Intervention

#### Functional Brain Activation

Recently, Perdue et al. (2022) conducted a quantitative meta-analysis using seed-based d mapping (Albajes-Eizagirre et al., 2019) on changes in brain activation pre/post reading intervention in people with—or at risk for—RD. In sum, eight fMRI studies that met predefined inclusion criteria (total aggregated sample size = 151 participants, mean age per study = 5.6–44 years) were included in the meta-analysis, which followed the strict PRISMA statement for transparent reporting of systematic reviews and meta-analyses evaluating the effects of interventions (Page et al., 2021). Intervention duration lasted from three to twelve weeks and various (in part commercially available) training programs were used, aimed at different reading component skills (e.g., phoneme awareness, morpheme-based spelling, grapheme-phoneme conversion, or reading fluency).

No statistically significant brain effects of reading intervention could be observed in this meta-analysis. According to the authors, one possible explanation could be the small set of included studies due to the exclusion of studies for methodological reasons. Additionally, even the studies that met the inclusion criteria suffered from small sample sizes. The primary limiting factor, however, is the use of region/volume of interest (ROI/VOI) analysis instead of whole-brain analysis, which renders objective coordinate-based meta-analysis difficult if not impossible and therefore has been a methodological exclusion criterion. Discussing their findings, Perdue et al. (2022) suggest, that future reading intervention studies should employ exploratory, spatially unrestricted whole-brain analysis in larger samples to adequately assess the effects of reading intervention on brain activation.

Furthermore, Barquero et al. (2014) reported an activation likelihood estimation (ALE) meta-analysis with a slightly different set of eight fMRI studies (seven studies with children and adolescents and one study with adults, total aggregated sample size = 173 participants) assessing functional activation patterns after reading intervention. Across the included studies, intervention periods ranged from 3 weeks up to two school years. As in the Perdue et al. (2022) meta-analysis, various different intervention programs were administered in the single studies.

Increased activation in RD participants following reading intervention was observed in the following brain regions of the typical reading network: left thalamus, right insula/IFG, left IFG, right posterior cingulate gyrus, and left middle occipital gyrus. In conclusion, and similar to the previously discussed meta-analysis, the authors note that the results must be interpreted with caution due to several methodological limitations at this relatively early stage of research, such as the high degree of heterogeneity in data acquisition and analysis methodology across studies and the generally limited number of published studies.

Despite the slightly disappointing and inconclusive meta-analytic (null-) results, a systematic qualitative review of reading intervention studies—also including MEG studies, which could not be part of the coordinate-based meta-analysis due to methodological reasons—essentially showed the following findings: fMRI and MEG studies identified pre-to-post changes in (a) the typical reading network as detailed in section “The Functional Neuroanatomy of Reading and Reading Disability”, thus indicating normalization of functional activation in RD and (b) additional cortical, sub-cortical, and cerebellar regions usually not included in this network, probably associated with compensatory reading mechanisms (Perdue et al., 2022).

In particular, multiple studies reported elevated levels of activation following reading intervention in the left hemisphere reading areas (Shaywitz et al., 2004; Richards et al., 2006b; Horowitz-Kraus et al., 2014; Heim et al., 2015). Importantly, this indicates that—through specific training—functions of the typical reading network can recover in people with RD. Additionally, initial group differences in activation levels between RD and typically developing controls were normalized in some studies, that is, differences before intervention were no longer detectable after intervention (Aylward et al., 2003; Richards et al., 2006a; Meyler et al., 2008).

In some of these studies, this normalization of functional activation also involved increases in the right hemisphere and sub-cortical regions (e.g., Meyler et al., 2008; Gebauer et al., 2012; Nugiel et al., 2019; Partanen et al., 2019). Equal levels of right-hemispheric activation in RD following reading intervention—compared with typical readers—could indicate a shift toward the typical engagement of these regions. Previous literature instead largely suggested that such changes may reflect compensatory processes, in the sense that people with RD engage regions outside of the typical reading network in order to make up for their deficits.

Across studies, the most consistent normalization effects could be observed in the right IFG (Temple et al., 2003; Meyler et al., 2008; Odegard et al., 2008; Horowitz-Kraus et al., 2014; Partanen et al., 2019). The right IFG is already activated during reading and reading-related processes before intervention and people with higher initial activation showed greater engagement after intervention (Hoeft et al., 2011). Functionally, the right IFG is thought to support articulatory recoding, working memory, and attention during reading (Shaywitz et al., 2002; Hancock et al., 2017).

Neuroplasticity associated with reading intervention in the right hemisphere was also identified in homologous regions of the left hemisphere reading network, that is, STG, OT cortex, and inferior parietal lobule (IPL) (Perdue et al., 2022). The exact functional role of greater activation following reading intervention in these right hemisphere sub-components, however, still remains unclear (for an in-depth discussion on this topic see Perdue et al., 2022). To conclude, contrary to previous findings, newer studies suggest that enhanced right-hemispheric activation in RD following reading intervention might reflect normalization rather than compensation.

#### Gray and White Matter Structure and Connectivity

With respect to GM volume, structural changes related to reading intervention in children with RD were identified in hubs of the typical reading network, sub-cortical and right hemisphere regions. This included increases in GM volume relative to the pre-intervention assessments in the left anterior OT cortex extending into the hippocampus, bilateral precuneus, right hippocampus, and right cerebellum (Krafnick et al., 2011). After an 8-week period without intervention, these effects were stable and an additional cluster of GM volume increase was identified in the right caudate.

Romeo et al. (2017) investigated neuroplasticity by means of cortical thickness in 65 children with RD (aged 6–9 years) of which *n* = 40 participated in a summer reading intervention program, that lasted for 6 weeks. The remaining *n* = 25 children constituted the waiting-list control group. A commercial multisensory program (centered on orthographic and phonological processing) was used. Results showed that the intervention group maintained their reading scores, whereas the waiting control group decreased in performance. On an individual basis, children who improved their reading scores—in the intervention group—had lower socioeconomic backgrounds than children that declined in reading performance. Comparing responders with non-responders, greater change in cortical thickness could be observed in responders in the following regions: bilateral middle-inferior temporal cortex, IPL, precentral cortex, and paracentral/posterior cingulate cortex, right STG and insula, and left MTG.

Regarding WM structure and connectivity, neuroplasticity associated with reading intervention could be observed in a number of studies (Perdue et al., 2022). Specifically, several DTI studies reported changes in structural connectivity and WM integrity linked to enhancement of reading performance after intervention (Davis et al., 2010; Richards et al., 2017; Huber et al., 2018). Increased FA and decreased mean and radial diffusivity might indicate that WM pathways increased in efficiency by improving communication among distant cortical and sub-cortical structures involved in reading.

Impressively, structural changes already occurred after only 2–3 weeks of intervention (same program as in Romeo et al., 2017) when children aged 7–12 years were scanned multiple times over the period of 8 weeks (Huber et al., 2018). Therefore, the brain delivers a fast adaptation response following the high demands of intensive training, i.e., detectable neuroanatomical rewiring processes as a consequence of reading intervention. The links between reading skill improvement and WM microstructure deviated from typical developmental trajectories during intervention. Consequently, this does not support the assumption of neuroanatomical normalization as reported in some functional activation studies. Study designs similar to the one employed by Huber et al. (2018), however, are costly and therefore rarely used, even though they provide important insights into the temporal progress of ongoing brain changes.

Davis et al. (2010) reported that changes in structural connectivity in response to a small group reading intervention (duration = 17 weeks) in eleven first graders (mean age = 7.5 years) were consistent with behavioral changes. Moreover, associations between functional connectivity and WM structure (Richards et al., 2018), together with incremental changes in WM microstructure during reading intervention as described before provide valuable insights into possible mechanisms of neuroplasticity in brain networks that enhance reading. Essentially, neural optimization in terms of rewiring of network connections might be related to the establishment of stronger brain circuits on the one hand and the reduction of inefficient connections on the other hand. Therefore, the strict distinction between neuroanatomical normalization vs. compensation mechanisms may not apply in these studies.

#### Functional Connectivity and Integration

Evidence regarding altered functional connectivity following reading intervention suggests that integrating dispersed functional networks facilitates reading improvements in RD (Perdue et al., 2022). Intervention-related neuroplasticity effects were found both during task-based and resting-state fMRI in diverse brain systems including fronto-parietal and cingulo-opercular networks (Horowitz-Kraus et al., 2015; Richards et al., 2016, 2017), and among low-level visual, dorsal attentional, and executive function networks distributed in various brain regions (Horowitz-Kraus et al., 2019).

Specifically, Horowitz-Kraus et al. (2019) examined changes in functional connectivity during task-based fMRI using a lexical decision task. They compared three groups (*n* = 18 each): RD, comorbid attention-deficit and hyperactivity disorder and RD (ADHD + RD), and typically developing (TD) in a computer-based intervention program targeting reading skills and executive functions, which lasted for 4 weeks. Independent component analysis was used to extract networks for connectivity analysis. Across the three groups, results showed positive correlations between reading speed gains and both increased functional network connectivity between the executive function component (bilateral superior frontal gyri) and the low-level visual component (bilateral FFG) and increased functional connectivity between the dorsal attention component (bilateral precuneus/posterior cingulate) and the low-level visual component.

In contrast, Richards et al. (2018) also found decreases in local functional connectivity following a computerized program focused on reading and writing (duration = 18 lessons). The sample consisted of *N* = 42 students (mean age = 11 years, 10 months). For example, during a multi-sentence reading comprehension task, local functional connectivity in the right middle frontal gyrus decreased in two RD groups, whereas it increased in a dysgraphia and a TD group. The above-reported findings were interpreted as reflecting modulation of attention-linked networks during reading. Since both increases and decreases in functional connectivity were observed, one could argue that this pattern reflects a process of re-adjustment toward an optimal level of integration and separation within and between different functional brain networks.

## Limitations, Open Issues, and Future Perspectives

Studies on the brain mechanisms underlying reading improvements following behavioral intervention for RD have provided tremendously valuable insights into the neurobiology of typical and atypical reading development. Taken together, however, there is only limited consistency across studies regarding possible neuroplasticity effects, as illustrated by the absence of (or only weak) meta-analytic evidence (Barquero et al., 2014; Perdue et al., 2022). Reasons for this heterogeneity of results are discussed below.

Meta-analyses are generally limited in scope due to strict inclusion/exclusion criteria. This is particularly evident in meta-analyses of brain effects. Specifically, in the recent meta-analysis by Perdue et al. (2022), 31 out of 39 thematically relevant primary studies had to be excluded because of ineligible imaging modalities, regionally restricted analysis strategies, imaging time points, and other methodological considerations. In addition, even the included studies used a variety of different fMRI activation tasks and methodological parameters for image preprocessing and statistical analysis, and generally suffered from small sample sizes, thus increasing the probability of both false positive and false negative results (Button et al., 2013).

The next issue concerns the participants in these studies themselves. In the reviewed studies, participants differed in terms of several aspects known to have an influence on reading development, such as age, home literacy environment, socioeconomic status, and initial skills. With respect to age, Suggate (2010) reported an interaction between grade at intervention and focus of intervention. In earlier grades, greater effects were elicited by phonics training, whereas in later grades, greater effects were elicited by comprehension training. Orthographic depth of the written language may also play a considerable role in this regard (e.g., Paulesu et al., 2001; Richlan, 2014, 2020; Martin et al., 2016).

In addition, there is the potential problem of (mis-) diagnosis and comorbidity. In particular, RD is often comorbid with atypical or delayed oral language development (Catts et al., 2009; Peterson et al., 2009), writing disabilities, ADHD, and math disabilities (e.g., Landerl and Moll, 2010; Willcutt et al., 2010). This, together with the generally large inter-individual differences with respect to responsiveness to reading intervention, may lead to higher variability of (potential) neuroplasticity effects, which, in turn, may lead to weaker meta-analytic results.

There is no clarity about whether specific regions or patterns of activation are required in order to provoke improvements in reading ability in RD. Numerous studies have shown effects within the typical reading network as well as outside. The differentiation between “normalization” vs. “compensation” effects is more complex than detecting activation in certain brain areas because multiple brain regions linked to the typical reading network are associated with other, more general cognitive networks as well (e.g., attention and executive function networks).

Future studies should try and identify networks of activation in addition to fundamental structural changes linked to improvement in reading ability. Furthermore, several factors regarding individual differences and interventions should guide research on the neural mechanisms of reading intervention. One way of providing more thorough evidence would be via longitudinal studies with a longer time frame (i.e., going beyond sole pre-/post-intervention assessments). Although extremely expensive and challenging to conduct, such studies would be desperately needed (Chyl et al., 2021).

Another desirable and extremely worthwhile approach would be to conduct multi-center studies with sufficient sample sizes, where the same methodologies are applied in a concerted and standardized effort. For example, Paulesu et al. (2001) investigated cultural differences across people with RD in the course of a cross-European PET project, whereas Jednoróg et al. (2015) conducted a large-scale multi-center, multi-language VBM study. Recently, another study showed that brain-wide association between inter-individual differences in brain structure or function and complex cognitive or mental health phenotypes, such as reading disability and its remediation, requires thousands of individuals (Marek et al., 2022).

Last but not least, the general issue of publication bias (i.e., withholding null findings and publishing statistically significant results) might create a false impression of substantial and reliable brain changes linked to reading intervention. This could explain some of the observed contradictory findings between studies reviewed here. To summarize, systematic and robust neuroplasticity effects in response to reading improvements across many studies could not yet be found. Therefore, further (pre-registered) research on the interplay between behavioral reading intervention and the brain mechanisms underlying typical and atypical reading is needed.

## Conclusion

In recent years, outstanding progress has been made in understanding the functional neuroanatomy of typical reading, RD, and reading intervention for RD. Our review of studies suggests that enhanced activation in right-hemispheric homologous regions of the typical left hemisphere reading network following behavioral intervention might reflect functional neuroanatomical normalization rather than compensation of brain mechanisms for reading. With respect to rewiring of white matter network connections in response to intervention, neural optimization might be related to both, the establishment of stronger brain circuits and the reduction of inefficient connections in RD.

Nevertheless, the field suffers from a lack of consistent neuroplasticity effects associated with improvement in reading ability across studies. Future studies should examine inter-individual differences and developmental trajectories more closely over a longer time frame. Additionally, the common dichotomy between “normalization” vs. “compensation” seems to be insufficient to explain the complex underlying neurobiology and a more integrated view of the brain mechanisms related to reading intervention should be employed.

## Author Contributions

JB and FR conceived and wrote the manuscript. Both authors contributed to the article and approved the submitted version.

## Conflict of Interest

The authors declare that the research was conducted in the absence of any commercial or financial relationships that could be construed as a potential conflict of interest.

## Publisher’s Note

All claims expressed in this article are solely those of the authors and do not necessarily represent those of their affiliated organizations, or those of the publisher, the editors and the reviewers. Any product that may be evaluated in this article, or claim that may be made by its manufacturer, is not guaranteed or endorsed by the publisher.
